# Impact of a culturally adapted digital literacy intervention on older people and its relationship with health literacy, quality of life, and well-being

**DOI:** 10.3389/fpsyg.2024.1305569

**Published:** 2024-04-15

**Authors:** Claudia Marisol Carrasco-Dajer, Aldo Renato Vera-Calzaretta, Silvia Ubillos-Landa, Juan Carlos Oyanedel, Virginia Díaz-Gorriti

**Affiliations:** ^1^Universidad Andres Bello, Programa De Doctorado En Educación Y Sociedad, Facultad De Educación Y Ciencias Sociales, Santiago, Chile; ^2^Departamento Ciencias De La Enfermeria, Facultad De Medicina, Universidad Catolica De La Santisima Concepcion, Concepción, Chile; ^3^Departamento de Kinesiología, Facultad Ciencias de la Salud, Universidad de Atacama, Copiapó, Chile; ^4^Facultad Ciencias de la Salud, Universidad de Burgos, Burgos, Spain; ^5^Departamento de Psicología Social, Universidad del País Vasco, Donostia-San Sebastián, Spain

**Keywords:** digital literacy, health literacy, quality of life, older people, well-being, culturally tailored intervention

## Abstract

**Introduction:**

Older people are the group with the greatest digital gap, so their digital literacy is important to improve the conditions in which they age.

**Methods:**

A study was conducted with pre- and post-evaluation of a digital literacy (DL) intervention in people aged 60 years and over. A total of 56 participants (experimental group *N* = 32 and control group *N* = 24) were recruited for convenience in community centers. The intervention was adapted to the needs of the participants, there were five face-to-face sessions and remote reinforcement for three months, carried out by trained university students for five months. Sociodemographic variables such as self-perception of socioeconomic level and education, among others, were evaluated. The impact was assessed using the digital literacy scale (MDPQ16), indicators of frequency and types of internet and mobile phone use, health literacy (SAHLSA and NSV), quality of life (SF-12), hedonic well-being (Diener’s SWLS and Cummins’ PWI) and perceived social support using the Zimet scale.

**Results:**

The intervention had a significant impact with an effect size of *r* = 0.27 on digital literacy, separate t-test comparisons revealed a markedly significant change for digital literacy in the experimental group, before and after the pre-post *t*-test_(31) =_ 3.56, *p* = 0.001, but not in the control group, *t*_(23)_ = 0.082, *p* = 0.93. No direct impact on health literacy, health-related quality of life, and hedonic well-being was identified. We examined the indirect impact of change in digital literacy and found that it correlated with improvements in well-being and social support, as well as quality of life. Individuals with significant changes were detected and compared with those who did not change.

**Discussion:**

Evaluation that contributes by identifying elements for improvement in future interventions and discusses the importance of culturally adapting continuing education in older people.

## Introduction

Population aging is an accelerated global reality (United Nations Organization, 2023, p. IV). This trend is pronounced in Latin America and the Caribbean, including Chile (National Institute of Statistics of Chile, 2023; Pan American Health Organization and Economic Commission for Latin America and the Caribbean, 2023, pp. 7, 81). At the same time, the digitalization of aging societies has generated a digital divide between older people (OP) and other age groups, a phenomenon that has been studied in various studies (Roque and Boot, 2016; Cardozo et al., 2017; Pontificia Universidad Católica de Chile y Caja de Compensación Los Andes, 2019; Sunkel and Ullmann, 2019; Ngiam et al., 2022).

Specifically in Chile, this gap is manifested in the fact that only 32% of OP use and have access to technologies, leaving two-thirds of this group in a situation of digital exclusion (Subsecretaría de Previsión Social del Gobierno de Chile, 2018). This problem is aggravated by economic and cultural factors, mainly affecting those in lower socioeconomic levels (Friemel, 2016; Mizrachi et al., 2020; World Bank, 2021). Consequently, a social fracture is created that increases inequality and the risk of social exclusion (Hasan and Linger, 2016; Cardozo et al., 2017).

In Chile, OP access to and use of technology, including the internet, computers, and mobile phones, is positively associated with educational level and inversely with age, with the mobile phone being the device most used by this group (Subsecretaría de Previsión Social del Gobierno de Chile, 2018). Against this backdrop, the development of Information and Communication Technologies (ICTs) is presented as an opportunity to increase access to information and social interaction, promoting new forms of social relations, such as social networks, which facilitate the social integration of the elderly (Cardozo et al., 2017; Castilla et al., 2018). However, these advances also bring with them challenges, such as the high speed of technological innovation and smartphone applications that are not always accessible (ChePa et al., 2023) and understandable to OP (Castilla et al., 2018).

Faced with these challenges, Chile has developed interest in digital transformation strategies, such as “Chile Digital 2035,” which emphasizes the digital literacy (DL) of OP. This strategy focuses on identifying OP as one of the priority groups and seeks to facilitate their adaptation to new technologies, especially in the field of health, thus contributing to their health and psychosocial empowerment (Paramio et al., 2015; Economic Commission for Latin America and the Caribbean, Republic of Chile-Senado, 2023).

### Digital literacy interventions in the elderly

The development of digital literacy programs for OP is based on a concept that has evolved since the United Nations Educational Scientific and Cultural Organization (UNESCO) is initial definition of “literacy” in 1958 (Organización de las Naciones Unidas para la Educación, Ciencia y Cultura, 1958), now integrating aspects of health (Galán and Zamora, 2015) and digital skills (Organización de las Naciones Unidas para la Educación, Ciencia y Cultura, 2019). Digital literacy is defined as the set of skills for operating digital devices and essential skills in Information and Communication Technologies (Friemel, 2016), and the skills and knowledge necessary to function in the information society (Martí et al., 2008).

Most studies on DL in OP come from developed countries, showing a preponderance of female participants, ranging in age from 55 to over 70 years, and the number of participants ranging from 39 to more than 100 (Ferreira et al., 2014; Chiu et al., 2016; Hasan and Linger, 2016; Castilla et al., 2018; Jimena, 2020; Lee et al., 2022; Ngiam et al., 2022; Sriwisathiyakun and Dhamanitayakul, 2022). Key components for program effectiveness are identified, such as theoretical underpinning, clear and measurable objectives, and preference for multifaceted interventions (Mirmohammadkhani et al., 2020; Pourrazavi et al., 2020; Stormacq et al., 2020). In addition, the importance of prior assessment of digital competencies and the adaptation of programs to the educational and cultural needs of PMs is emphasized (Paramio et al., 2015; Roque and Boot, 2016; Ghorbanian and Nikou, 2021; Haase et al., 2021; Shi et al., 2021; Kanakaris and Pavlis, 2022).

DL sessions range from 6 to 8, with durations of 1 to 2 h, and are held in collective and individual formats. Personalized, volunteer-developed interventions that combine synchronous and asynchronous formats show better results, allowing PMs to progress at their own pace (Ferreira et al., 2014; Jimena, 2020; Ghorbanian and Nikou, 2021; Arellano-Rojas et al., 2022; Ghorbanian et al., 2022; Kanakaris and Pavlis, 2022; Ngiam et al., 2022). Contents include use of social networks, personal development, self-sufficiency, and skills for searching for information and collaborative work online (Hasan and Linger, 2016; Castilla et al., 2018; Jimena, 2020). This data makes it possible to identify what is most used, however, there is not enough evidence to determine which is the most effective formula.

### Effects of digital literacy interventions on older persons

#### Increasing digital literacy levels

These DL programs focused on OP make it possible to reduce the digital divide (Valenzuela et al., 2022) as they increase their technological knowledge (Ma et al., 2020), significantly improve digital literacy scores in the intervention group compared to controls (Ngiam et al., 2022), contributing to the digital divide (Valenzuela et al., 2022). with the development of skills and use of devices such as mobile phones (Lee et al., 2022), digital tools for the detection of fake news, and quality information (Moore and Hancock, 2022).

#### Increases in health literacy levels

According to the World Health Organization, health literacy (HL) refers to the cognitive and social skills that motivate and enable people to seek, understand, and use health information to maintain good well-being (World Health Organization, 1998), a construct that includes not only the ability to read health information, but also, understanding concepts, interpreting medical instructions, and the ability to make informed health care decisions. Studies in this area indicate that OP have a low level of SA (Mirmohammadkhani et al., 2020; Stormacq et al., 2020), particularly in socioeconomically disadvantaged groups (Stormacq et al., 2020).

In addition, in the current context of technologization of social and health services, OP have lower levels of literacy, health literacy and digital literacy (Xie, 2011; Handtke et al., 2019; Ghorbanian and Nikou, 2021) requiring these skills to understand the information available on digital platforms, adequately solve a health problem (Norman and Skinner, 2006; Stormacq et al., 2020), and access quality services (Ghorbanian and Nikou, 2021; Shi et al., 2021; Sobral and Sobral, 2021; Ghorbanian et al., 2022; Lee and Tak, 2022; Sriwisathiyakun and Dhamanitayakul, 2022).

This deficit is associated with sociodemographic variables, cultural barriers such as lack of familiarity and fear of using ICTs (Xie, 2011; Handtke et al., 2019; Ghorbanian and Nikou, 2021), frequency of internet use and the possibility of learning how to use it to find health resources (Shi et al., 2021; Kanakaris and Pavlis, 2022). Thus, these evidence-reported factors would be classified as individual, interpersonal, and community, however, these results remain inconsistent (Shi et al., 2021), demonstrating a gap in the body of scientific knowledge in this area.

In this regard, experience of interventions reports mixed results, those that promoted the continuous use of digital devices demonstrated a decrease in fear of their use, an improvement in interest in and ability to handle ICTs (Castilla et al., 2018) and an increase in the use of digital health tools and services (Ghorbanian et al., 2022). Most showed effectiveness with respect to the impact on the ability to use health information, although on the competence to understand health information, they were shown to be ineffective (Stormacq et al., 2020). The results also suggest that health empowerment of older people by making them digitally literate is possible (Ghorbanian and Nikou, 2021; Ghorbanian et al., 2022) through different intervention methods (Ghorbanian and Nikou, 2021).

### Effects of DA programs on OP quality of life

The construct quality of life is increasingly being used in assessments within the health sector as a marker of well-being (Urzúa, 2010; Martínez and Gallardo, 2020). In addition, there is a growing interest in quantifying health-associated quality of life, which is defined as “the subjective assessment of the domains of your life that are perceived as important during a particular time” (Burke, 2001).

These programs can improve the quality of life of participating seniors by enabling them to access online services, such as shopping, virtual healthcare, government paperwork, and banking services (Mirmohammadkhani et al., 2020; Aggar et al., 2023), which can be significantly important depending on the mobility and self-esteem difficulties they suffer. Otherwise, it also improves physical health (Ghorbanian and Nikou, 2021; Shi et al., 2021; Sobral and Sobral, 2021; Sriwisathiyakun and Dhamanitayakul, 2022), favors active aging, finding that highly digital seniors have a better healthy life (Mizrachi et al., 2020), along with decreasing the negative effects of aging on health such as memory decline (Chan et al., 2016; ChePa et al., 2023) for example. In addition, greater use of the internet is associated with a better quality of life (Boz and Karatas, 2015). However, experiences of DL have also been reported in which there is no evidence of effects on the perception of health and quality of life (Lee et al., 2022), so there is no consensus on this.

### Effects of DA programs on perceived well-being

Considering the definition of subjective well-being as people’s evaluations of their own life, which can be judgments, such as life satisfaction (cognitive), feeling-based evaluations, including moods and emotions (emotional) (Pavot and Diener, 1993; Diener and Suh, 1997); regarding these components of psychosocial health of older people, digital skills deficits and poor understanding of health messages are related to adverse effects on well-being (Stormacq et al., 2020). Generally, OP who master new technologies have a good level of psychological well-being due to the feeling generated by being able to achieve it, which helps to improve their position in the eyes of their family and even in society (Hasan and Linger, 2016; Cardozo et al., 2017; Shi et al., 2021; Sobral and Sobral, 2021; Sriwisathiyakun and Dhamanitayakul, 2022; Aggar et al., 2023), in contrast, the secondary outcomes of Ngiam et al. (2022) included improvements, however, they were not statistically significant in the personal well-being score (Ngiam et al., 2022). In turn, Hasan and Linger (2016) report that the increase in digital skills increases social well-being in the dimensions of social participation and involvement, occupation, control over daily life and dignity. Likewise, greater use of the internet would be a predictor of higher levels of well-being and satisfaction with life (Heo et al., 2015).

### Effects of DA programs on perceived social support

Under the conceptual framework that defines social support as “the set of expressive or instrumental provisions – perceived or received – provided by the community, social networks and trusted individuals, provisions that can occur in both every day and crisis situations (Lin, 1986), it has been stated in the evidence that the development of technological skills and competences in this age group favors their social interaction (Shi et al., 2021; Sobral and Sobral, 2021; Sriwisathiyakun and Dhamanitayakul, 2022), helping to address social isolation and loneliness in PMs by applying various technologies such as ICTs, video games, robotics, personal reminder information and social management system, asynchronous pairs, chat support and telecare among others (Khosravi et al., 2016).

The development of digital interventions has been shown to have a positive impact on the perception of social support, according to a study carried out in Chile (2004), which attributed the positive results to the ability of older adults to become visible and receive recognition in their social environment thanks to the digital skills acquired (Cerda and Llaña, 2005). Likewise, other research currently confirms that the use of technology enhances social connection (Ma et al., 2020), since the rapid digitalization and technological revolution allows the integration of OP into society, since technology facilitates interaction (Pan American Health Organization, 2023) and the maintenance of relationships significant social and emotional factors, and social support (Van Volkom et al., 2013). Likewise, greater use of the internet would be a predictor of higher levels of social support (Heo et al., 2015) and is also associated with an unhealthy lifestyle (La Duplaga, 2021).

Notwithstanding what has been analyzed, the evidence described allows us to state that some of the studies of DL interventions were found to show enormous effect sizes of *d* = 2 or *r* = 0.60 (De Main et al., 2022), which could be defined as overestimates.

The present study describes an intergenerational digital literacy intervention that was developed and culturally adapted for OP in a territorial sector of the commune of Concepción-Chile and evaluates its effectiveness in health literacy (HL), quality of life (QoL) and well-being. As it is quasi-experimental research with pre- and post-intervention evaluations, it is a contribution because it allows us to identify improvements and increase the effects in subsequent experiences. In this way, it contributes with applied and updated evidence that guides the design of integrative systems capable of responding socially and technologically to demographic changes and the needs of OP.

The hypotheses put forward were:

*H1*: The intervention will improve the level of digital literacy, mobile phone and internet use in the group of participating OP compared to control.

*H2*: The intervention will improve the level of health literacy in the group of participating PMs compared to control.

*H3*: The intervention will improve QoL in the participating OP group compared to control.

*H4*: The intervention will improve well-being and social support in the participating OP group compared to control.

## Methods

### Design

A quasi-experimental non-equivalent control group study was conducted, with pre and post evaluation of a Digital Literacy intervention in the elderly. A culturally adapted multicomponent program was implemented, carried out in a non-formal education context in the community during the year 2022. After establishing the baseline, three months after the intervention, its effects on the increase in the use of technologies, the level of digital literacy and in health, health related QoL, social support and subjective well-being were evaluated.

### Participants

Elderly people from the city of. Concepción (Chile) participated in the study. The inclusion criteria were to be 60 years of age or older and to be members of 12 organizations in the urban sector that participated in community workshops. The exclusion criteria were to be inactive members of the community organizations or to have sensory difficulties that prevented them from answering the survey and participating in the intervention.

The final sample consisted of 56 older adults. Eighty-four percent were women, with an average age of 73 years. The greater participation of women responded to the fact that in this type of organization female participation is in the majority. On the other hand, 43.6% reported a basic or lower level of schooling and 67.6% rated their socioeconomic situation as fair or poor (see Table 1).

**Table 1:** Sociodemographic characteristics of the participants.

		*M*	*SD*
Age		73.0	6.3
		*N*	*%*
*Sex*			
	Woman	47	83.9
	Man	9	16.1
*Education*			
	Illiterate	1	1.8
	Basic	23	41.8
	Media	17	30.9
	Technique	6	10.9
	Superior	8	14.5
*Socio-economic situation*			
	Bad	7	12.5
	Regular	32	57.1
	Good	15	26.8
	Very good	2	3.6
*Group*			
	Experimental	32	57.0
	Control	24	43.0

### Formation of the groups

Two groups were formed with non-random assignment of subjects (experimental group *n =* 32 and control *group n* = 24). Participants were recruited from their own neighborhood center’s. Older people waiting to participate in the program made up the control group. They continued to develop community activities such as handicraft and dance workshops.

### Statistical power of the sample

With respect to sampling and statistical power, a review of the evidence identified that the effect sizes of some similar interventions are very high; more than one standard deviation or *r* = 0.50 or more (Xie, 2011; Kim and Xie, 2017). However, other estimates of the effect of digital literacy give effect sizes of *r* = 0.18 (Ghorbanian et al., 2022).

For this study, it was estimated that with an effect size similar to that of the training interventions (*r* = 0.20 or 0.21), with a statistical power of *α* = 0.05 and *β* = 0.80, 138 subjects were needed. However, in the present study only an *n* = 56 was achieved, which for an effect size of *r* = 0.21 indicates a statistical power of 0.41, below 0.80 which is desirable. To address this problem, which is common in research of this type, analyses were supplemented by the application of a constructed non-causal baseline design, i.e., comparing post-test treatment data with mass sample scales; and the use of reliable change scores (Páez et al., 1993).

### Variables and measuring instruments

In this study, predicted effects of participation in digital literacy formation were assessed: (a) digital literacy; (b) health literacy; (c) quality of life related to health and health behavior; (d) well-being; and (e) social support.

*Digital Literacy* measured with the Mobile Device Proficiency Questionnaire (MDPQ-16) (Roque and Boot, 2016). It is made up of 16 items that measure 8 dimensions: mobile device basics, communication, data and file storage, internet, calendar, entertainment, privacy, problem solving, and software management. The answer is on a 5-point Likert scale (1 = I’ve never done it to 5 = very easy). The Cronbach’s alpha of the original version is 0.99. In this study, the reliability of the scale in the pre-test was 0.91 and in the post-test it was 0.90. It also has good test–retest reliability, obtained by correlating the responses of the pre-test with the post-test, *r*_(55)_ = 0.697, *p* ≤ 0.001.

*Availability and use of technological devices and the internet access* with a series of self-perception and self-assessment questions along with a dichotomous 10-item scale on mobile phone use (pre-test: Cronbach’s alpha (α) = 0.75; post-test: *α = 0.76; test–retest =* 0.78, *p* ≤ 0.001); and another *15-item* questionnaire on internet use (pre-test: *α* = 0.86; post-test: *α* = 0.88; test–retest = 0.82, *p* ≤ 0.001). These scales were created by the team of researchers based on the 2018 National Quality of Life Survey of Older Adults in Chile (Subsecretaría de Previsión Social del Gobierno de Chile, 2018).

*Health literacy* was measured with the 6-item Newest vital sign or NSV-6 (Weiss et al., 2005) and the Short Assessment of Health Literacy for Spanish-speaking Adults or SAHLSA-50 (Monsalves et al., 2016). The NVS-6 measures the level of comprehension of instructions and the ability to perform numerical calculations on information on a nutrition label. It is used as an indicator of functional health literacy. In the validation for Chile, the reliability coefficient KR-20 was 0,7,478 (González et al., 2023). Cronbach’s alphas in this study were 0.57 in the pre-test and post-test. The test–retest index was 0.28, *p* = 0.048.

*The SAHLSA-50* consists of 50 items and assesses the ability to read and understand the common medical terms of a Spanish-speaking adult, in the validation for Chile, a Cronbach’s alpha of 0.92 was obtained (Lee et al., 2006). For this study, Cronbach’s alphas were 0.89 in the pre-test and 0.90 in the post-test. The test–retest index was *r* = 0.824, *p* = 0.0001.

*Health-related quality of life* measured with SF-12 with good reliability reported by previous studies with *α* = 0.90 (Martínez and Gallardo, 2020) and *α =* 0.74 (Vera et al., 2014). Cronbach’s alphas in this study were 0.61 in the pre-test and 0.85 in the post-test. The test–retest index was 0.24, *p* = 0.096.

*Subjective well-being* measured with the SWLS-5 scale, which evaluates the dimension of general life satisfaction with 5 items. The Cronbach’s alpha reported by its authors is 0.87 (Diener et al., 1985) and another subsequent study 0.856 (Ramírez and Lee, 2012) and 0.82 (Vera et al., 2012). In this study, the internal consistency indices were satisfactory (pre-test*: α =* 0.79; post-test: *α* = 0.78), although test–retest reliability was *r*_(55)_ = 0.222, *p* = 0.10.

*Personal well-being index* or PWI-8 was also used, an 8-item satisfaction scale with life domains (Cummins et al., 2003), which obtained satisfactory reliability in this study (pre-test: *α* = 0.90; post-test: *α* = 0.84), as well as a good retest test of *r*_(55)_ = 0.392, *p* = 0.003.

*Social support* measured with the 12-item Multidimensional Social Support Scale (Zimet et al., 1988) with a Cronbach’s alpha of 0.88 and subsequent study of 0.86 (Arechabala and Miranda, 2002). Reliability in this study was also satisfactory (pre-test: α = 0.87; post-test: α = 0.85), as was a good retest of *r*_(55)_ = 0.53, *p* = 0.0001.

*Sociocultural measures* a measure of self-perceived socioeconomic status (Bad, Fair, Good, Very Good), and a measure of schooling (Illiterate, Basic, Medium, Technical, Higher) were used as indicators. Information on sex (male, female) and age was also obtained.

### Procedure

To access the intervention subjects, through the municipal delegation of the city of Concepción, the leaders of different community organizations of the elderly were contacted, who facilitated the contact with the potential participants.

Before carrying out the intervention, once informed consent was obtained, each subject was interviewed to assess their needs and expectations regarding Digital Literacy (DL), the objective of which was to incorporate them into the design and implementation of each of the sessions contemplated in the protocol. In addition, questionnaires were applied to establish the baseline for the different variables of interest. Then, three months after the end of the intervention, a second interview was carried out to apply the questionnaires and thus obtain the post-measurement of the variables.

The implementation of the intervention was carried out by university students, digital natives, who were duly trained as literacy teachers. Throughout the intervention process, two nurses who are experts in community work and aging were supervised.

The implementation of the intervention followed a protocolized structure in planned activities based on the achievement of the objectives set according to the needs and expectations expressed by the participating, most of them focused on communication and leisure activities. To safeguard cultural sensitivity, awareness and respect for the aging process were encouraged, as well as for the values and beliefs of OP.

The sessions were held at the community headquarters of each organization.

At the end of the year, a ceremony was held to award the certification that accredited participation in the program.

### Intervention

The intervention consisted of a digital literacy program in a non-formal community educational setting. This was tailored to be culturally competent (Handtke et al., 2019). Furthermore, it was customized based on individual needs, preferences, and experiences, combining face-to-face training, and online follow-up. Furthermore, the design was based on Henderson’s 14 needs theory (Henderson, 1966), the solidarity and intergenerational transfer model (Sánchez et al., 2014; Jimena, 2020) and the older adult education or gerontology approach (Fernández, 1999).

The program consisted of a maximum of 5 practical sessions of 1 h each. In these sessions, the transfer of skills in the use of cell phones, tablets and personal computers was encouraged, according to the needs and interest expressed by the users. Two components were considered, one related to information (learning about technological communication devices), and the other focused on skills (safety, adaptability, use of applications and search for relevant information). The contents covered in each session are presented in Table 2.

**Table 2:** Contents of the sessions.

Session	Contents
0	It consisted of a telephone call made by each monitor to the PMs with the purpose of introducing themselves and knowing their needs and expectations, along with the characteristics of their mobile device and internet connectivity, with the aim of designing the work protocol.
1	Basic handling and safety of the device. Planning reinforcement activities to be carried out at home with remote supervision.
2	Internet access, sites of interest, search for information of interest on the sites. Planning reinforcement activities to be carried out at home with remote supervision.
3	Management of digital applications of your interest, basic tools of the application. Planning reinforcement activities to be carried out at home with remote supervision.
4	Management of digital applications of your interest, advanced application tools. Planning reinforcement activities to be carried out at home with remote supervision.
5	Closing the process by answering questions about the contents worked on in the previous sessions or other new emerging needs. Planning reinforcement activities to be carried out at home with remote supervision.

### Statistical analysis

A descriptive analysis of the sociodemographic and digital literacy variables of the sample was carried out, calculating means with standard deviations (SD), frequencies and percentages. The analysis of the internal consistency of the items of the scales used was performed with Cronbach’s alpha (Cronbach, 1951) and to evaluate their stability the Pearson’s correlation re-test was performed.

The study of the normality of the distributions of the variables was carried out with the Shapiro Wilk test and the homoscedasticity of the groups with the Levene test.

On the other hand, to evaluate the hypotheses, repeated measures analysis of variance was calculated before and after the test, comparing the experimental and control groups, to evaluate the effect of the treatment in interaction with time. Mean differences (*t*-test for paired samples) were analyzed to measure changes in group measures at baseline assessment and at 3 months post-intervention.

Using the calculation of the reliable rate of change (Jacobson and Truax, 1991), significant changes in the DL variable were examined individually, which allowed the identification of cases that worsened, remained the same, and improved significantly. Pearson’s correlation was performed between the DL change scores (MDPQ-16) with changes in SA, health-related QoL, social support, life satisfaction, and personal well-being.

### Ethical considerations

The research project was approved by the Scientific Ethics Committee of the Catholic University of the Most Holy Conception - Chile with the registration ORD 11/2022 which is governed by the Helsinki Convention (World Medical Association, 2017). Informed consent was applied before each evaluation, and personal data was safeguarded to maintain confidentiality and anonymity. At the end of the study, the results were presented to the participants and the interested parties were given sessions to reinforce the learning.

## Results

### Relationship of sociodemographic variables with the mastery and use of technologies

First, the sociodemographic profile of the people who had a greater mastery and use of technologies in the pre-test was analyzed. A significant and negative correlation was found between DL (*r* = −0.394, *p* = 0.003), mobile phone use (*r* = −0.472, *p* ≤ 0.001) and internet (*r* = −0.623, *p* ≤ 0.001) and age. The differences according to the level of schooling are also significant (DL: *F*_(2.52)_ = 8.784_,_ *p* ≤ 0.001; mobile phone use: *F*_(2.52)_ = 16.066, *p* ≤ 0.001; internet use: F_(2.52)_ = 9.234, *p* ≤ 0.001). People with a low level of schooling (DL: *M* = 25.50, *SD* = 13.36; mobile phone use: *M* = 3.46, *SD* = 2.38; internet use: *M* = 3.08, *SD* = 3.65) have a lower DL handle and a lower use of technologies than people with medium education (DL: *M* = 31.29, *SD* = 11.12; mobile phone use: *M* = 6.18, *SD* = 1.70; internet use: *M* = 6.29, *SD* = 3.55) and above (AD: *M* = 43.57, *SD* = 13.83; mobile phone use: *M* = 6.86, *SD* = 1.51; internet use: *M* = 7.86, *SD* = 3.16). Likewise, people with high education have a higher mastery of DL than those with medium education. Self-perception of socio-economic level was not associated with the variables of AD and use of technologies.

### Comparison of the experimental group and control in the pretest in the impact variables

To ensure the internal validity of the study, the similarity at baseline in the experimental and control group was analyzed. The ANOVA of the pretest showed that there were no significant differences between the two groups in the mean scores of the pretest of all the variables analyzed (for the pretest means see Table 3).

**Table 3:** Comparison between experimental group and pre-test and post-test control in digital literacy, health literacy, health-related quality of life, well-being, and social support.

	Experimental group				Control group						
	Pre-test		Post-test		Pre-test		Post-test		Interaction effect		
Variables	*M*	*SD*	*M*	*SD*	*M*	*SD*	*M*	*SD*	*F*	*p*	η_p_^2^
Digital literacy (DL)	29.9	13.8	36.3	15.2	34.5	1.2	34.8	14.9	4.119	0.047	0.071
Mobile phone use	4.8	2.7	5.1	2.5	5.6	2.1	5.8	2.4	0.009	0.925	0.000
Use of the internet	4.9	4.2	5.0	4.2	5.8	3.6	5.7	4.0	0.193	0.662	0.004
Health literacy (NSV)	1.6	1.3	2.1	1.5	1.8	1.4	2.3	1.3	0.001	0.980	0.000
Health literacy (SAHLSA)	42.3	6.3	43.3	6.5	44.3	5.3	44.6	5.3	0.375	0.543	0.008
Social support	35.5	8.9	37.1	9.6	33.6	9.6	35.5	7.1	0.010	0.920	0.000
Life satisfaction SWL 5	28.7	4.4	26.0	6.3	27.0	6.2	27.3	5.2	2.692	0.107	0.047
PWI8 personal wellness	60.0	12.2	61.0	11.2	62.1	13.0	63.5	10.1	0.011	0.915	0.000
SF-12 quality of life	30.0	4.5	31.7	8.4	27.6	5.2	30.5	6.4	0.230	0.634	0.005

### Evaluation of the impact of the program by comparing the experimental group with the control group

To examine the hypothesis, a repeated measures ANOVA comparing experimental and control group for dependent variable (e.g., digital literacy and so on) was carried out.

The analysis of repeated measures shows a significant time effect on the DL variable (*F*_(1.54)_ = 4.701, *p* = 0.035, η_p_^2^ = 0.080 and observed power = 0.57). The effect of the interaction of comparisons of pretest scores with posttest DL scores is also significant (*F*_(1.55)_ = 4.119, *p* = 0.047, η_p_^2^ = 0.071 and unilateral observed power = 0.64). There has been a significant increase in knowledge and skills of digital technologies in the experimental group compared to the control group. In the rest of the variables, the effect of interaction is not significant (see Table 3).

Separate t-test comparisons revealed markedly significant change for DL dependent variable in the experimental group, *t*-test pre-post *t*_(31) =_3.56, *p* = 0.001, but not in the control group, *t*_(23)_ = 0.082, *p* = 0.93.

Regarding the first hypothesis, this was supported by the direct effect of the intervention increasing the level of DL in the participants, with an effect *r* = 0.27 translating the eta square value into a correlation. On the other hand, making a biserial point correlation with pre and post changes (post score minus pre, the higher the score, the greater the improvement) with intervention (if = 1 and no = 0) the same similar effect of *r* = 0.27 was found. However, hypotheses 2, 3 and 4 were disconfirmed by the results, as the experimental group showed no improvement in HL, QoL linked to health, well-being, and social support.

### Evaluation of the program comparing post-test experimental group with a constructed non-causal baseline

To confirm the results obtained with a sample that meets the statistical power requirements of *N* = 138, the procedure called “Constructed non-causal baseline” was applied, where the means and standard deviations obtained by the experimental group of this study in MDPQ-16 (DL) in the post-test (*N* = 32; *M* = 36.3; *SD* = 15.2) with those of a large sample of older people in the United States (Roque and Boot, 2016) (*N* = 105; *M* = 20; *SD* = 11). The experimental group t-test versus the U.S. study showed significant differences, *t*_(136)_ = 5.47, *p* = 0.0001, *d* = 1.47 and *r* = 0.55. Likewise, the differences between the experimental group and the Chilean study are marginally significant: *t*_(183)_ = 1.09, *p* = 0.10, *d* = 0.21. In both cases, the experimental group shows more knowledge and skills in DL than the two baseline groups, although the effect sizes are large in the case of the comparison with the USA and small in the case of Chile.

### Reliable change assessment

The reliable change score of Jacobson and Truax (1991) or CR index (Iraurgi, 2010) was applied. The RCI standardized change score is the absolute difference required for a change score to be considered reliable or greater than the change due to measurement error.

The steps to calculate RCI are as follows:

1. Calculate standard measurement error SEM = s√1-rxx. s is DT from a reference group or large sample or global pretest DT
  rxx fiabilidad Chronbach’s alpha o test retest
2. Calculate SDIFF
  SDIFF = √ 2*(SEM* SEM or SEM2)
3. Calculate individual change score Diff = xt2 –xt1.
  The higher the score, the higher the positive variable.
4. Calculate RC = xt2 –xt1 /SDIFF
  RC is equal z-score example MDPQ
5. If CR ≥1.96 (standard value) or greater than 5% distribution - no error should be measured only.

For PMDQ in this studio

1. variability s = 14.44 Chronbach’s α = 0.91
  SEM = 14.44 √1–0.91 = 0.3
  14.44 × 0.3 = 4.33, i.e., SEM = 4.33
2. SDIFF = √ 2(4.33*4.33) = √ 2(18, 76)
  SDIFF = √ 2(18.76) = √ 37.51 = 6.12
3. In a case you present: xt1 = 25 xt2 = 55 Diff = 30
4. 30/6.12 = 4.9
5. 4.9 > 1.96 = Reliable Gearbox.

Table 4 presents results of the reliable change indicate that 7 out of 32 people improved significantly in the treatment group, i.e., 21.9%. The rest improve, but do not exceed the cut-off point of 1.96. In the control group, 4 out of 24, or 16.7%, improved due to autonomous learning, 15 (62.5%) did not change, and 5 (20.8%) worsened significantly. These results make it possible to detect failed intervention groups to reinforce them, such as cases 28, 30, 33 and 38 that improve less than 0.50, as well as to interview those that improve to understand good practices, such as subjects 40, 46 and 48 (Table 4).

**Table 4:** Reliable change results for each case.

Group	Cases that improve	Cases that remain	Cases that get worse
Experimental (*n* = 32)	22, 29, 37, 40, 46, 47, 48.	23, 24, 25, 26, 27, 28, 30, 31, 32, 33, 34, 35, 36, 38, 39, 41, 42, 43, 44, 45, 49, 50, 51, 52, 53.	
Control (*n* = 24)	6, 7, 8, 14	1, 2, 3, 4, 5, 10, 11, 12, 13, 15, 16, 19, 21, 54, 55.	9, 17, 18, 20, 56
Total (*n* = 56)	11	40	5

### Correlation between reliable change in digital literacy and change in the other variables in the experimental group

Reliable change scores in DL were correlated with change scores (post-test minus pre-test, the higher the score, the greater the improvement) in SA, QoL links to health.

The reliable change in CDMD trendily correlated with the improvement in health-related QoL assessed by SF-12, *r*_(29)_ = 0.25, *p* = 0.09, significantly with the improvement in well-being, assessed by SWL *r*_(30)_ = 0.33 *p* = 0.039, in the same sense but not significantly with the well-being assessed by the PWI *r*_(30)_ = _._21, *p* = 0.14, and with a tendency to improve social support *r*_(25)_ = 0.27 *p* = 0.10.

## Discussion

The relevance and development of digital literacy in the elderly make it a propitious scenario to analyze the impact of the intervention programs and methods used. Considering that technology education is largely seen as a crucial element for the effective use of technologies (Van Volkom et al., 2013; Heo et al., 2015; Cardozo et al., 2017; Castilla et al., 2018; Economic Commission for Latin America and the Caribbean, Republic of Chile-Senado, 2023). However, there is little research in developing countries that has focused on identifying the procedures implemented and their effects on the adoption of technology by older people. Therefore, this study investigated the effects of an intergenerational and culturally adapted literacy intervention on health and psychosocial variables, providing evidence that will facilitate future experiences.

The results of this study showed that the participants with the highest level of DL and greater use of technologies such as mobile phones and the internet are the youngest participants with a higher level of education, unlike what was found by Ferreira et al. where educational level was not a related factor (Ferreira et al., 2014).

The results showed a direct effect of the intervention on the improvement of the DL level with a significant effect size of *r* = 0.27, confirming hypothesis 1. Despite the fact that the power of the sample was low and the results of the effect size were smaller than those described in some studies (Roque and Boot, 2016), the efficacy of the intervention was confirmed – although the evaluation using paired samples from the sociocultural context showed a smaller effect, results similar to those presented by Valenzuela et al. (2022).

The results of reliable change showed that the percentage of people who improve is higher in the experimental group than in the control. Similarly, while among the OP who have received the intervention, none worsens, one-fifth of the control group worsens. Results that are consistent with the results of Ngiam et al. (2022), and Ma et al. (2020) which reflected the significant improvement in digital literacy in those who are intervened in contrast to controls, also coincides with the systematic review of Ghorbanian et al. (2022) and the Chilean study of Ferreira et al. (2014).

In relation to the results of mobile phone and internet use, no significant improvements are seen when comparing both groups, contrary to the conclusion of Lee et al. who show that this type of intervention improves the use of devices such as the mobile phone (Lee et al., 2022) and the internet (Heo et al., 2015). In addition, increasing internet use is a significant predictor of higher levels of social support and greater life satisfaction and psychological well-being among older adults (Heo et al., 2015), which could help explain the results presented below.

The direct effect is not observed in SA, well-being, and social support, suggesting that mere DL does not expand to improvements in these variables. This could have been affected by several exogenous variables, so it is important to remember that the control group was not passive, but active: the people who made it up did not participate in the DL but continued to attend a weekly workshop at the community center, so this social integration activity probably helped to maintain well-being and to perceive social support.

On the other hand, the association between the change scores in DL and the impact variables in the experimental group shows that there are indirect effects on personal well-being and social support. However, this indirect effect did not occur in HL. Cases with substantial improvement in DL (high change score) were characterized by improvements in well-being and, to a lesser extent, in social support, coinciding with Ma et al. (2020) and in QoL. However, there was no direct effect of the intervention on QoL, with no significant difference between the two groups, which coincides with the results of Lee et al. (2022) and contrasts with the results of Boz and Karatas (2015).

Carrying out this type of constructive evaluation of interventions carried out in the field of AD in PM allows for improved treatment, as it facilitates the detection of reliable improvements and worsenings. By interviewing these cases, it is possible to consider the improvements to be made in future DL interventions in OP, inferring good and bad practices. Allowing us to conclude how to strengthen and improve the intervention and its effects on DL and HL.

Important limitations of the study are self-selection, the application of a quasi-experimental design, and the fact that some subjects did not perform all sessions. The selection bias occurred when the OP of the indicated organizations was invited to participate, and the interested parties formed the intervention group and the non-interested ones formed the control group, so in future studies it will be advisable to select with a control group that does not participate in any community organization. At the same time, the assignment of OP in both groups should be randomized to ensure greater internal validity. Also, the inclement weather associated with the winter limited the participation of some of the people in the intervention group, which prevented them from carrying out all the sessions and influenced the loss of cases. Evaluating the quantity and quality of sessions held is important.

It should be considered that the intervention did not contemplate specific content of physical HL, and this should be incorporated, since it was found that the intervention had no effect of any kind on HL. Positive mental health reinforcement and social integration activities should be integrated. OPs are more receptive to improving well-being than focusing on chronic disease management. Sutipan et al. (2017) reviewed eight articles in five different countries, including Spain (*n* = 2), the United Kingdom (*n* = 1), Hong Kong (*n* = 1), Taiwan (*n* = 1), and the Netherlands (*n* = 1) on the impact of positive psychological interventions on the well-being of healthy OP, finding that most were effective. Two studies with an OP well-being intervention program, in China and Spain, evaluated the intervention and control group before and after, finding that interventions such as the thank-you visit, doing three good things, doing three fun things, and using distinctive strengths in a new way, increased well-being (Proyer et al., 2013; Sarrionandia et al., 2022). Therefore, interventions of this type of improvement of well-being would have an impact on HL or positive mental health.

Another fundamental element that should be considered to improve the sensitivity and effectiveness of the intervention is related to the variables included in the evaluation. These variables should integrate areas that are of interest and meaning to OP, and that allow them to reflect their experience in meeting needs and expectations when participating in this type of program (Kanakaris and Pavlis, 2022), such as self-efficacy in health and digital (Ghorbanian et al., 2022). Finally, implementing a program to reinforce the learning and skills achieved with the intervention will contribute to maintaining the effects on the participating OPs.

## Conclusion

The effects of multicomponent, culturally adapted DL intervention in OP showed that it is possible to achieve changes with medium direct impact on DL, and indirect impact on mental health or well-being. No direct or indirect effects on HL were identified. These types of studies contribute to the reduction of the digital divide and are a contribution that supports future interventions that should integrate content related to the improvement of health and well-being, evaluate self-efficacy, and add learning maintenance sessions, as well as generate spaces for intergenerational solidarity exchange that promotes the social integration of the various age groups.

Finally, digital literacy programs for older people in countries such as Chile are essential to promote social inclusion, improve the conditions in which they age and empower them in an increasingly technologized context, and in this way, they contribute to reducing the digital divide and facilitating the way for older people to fully enjoy the opportunities offered by technological development.

## Data availability statement

The original contributions presented in the study are included in the article/supplementary material, further inquiries can be directed to the corresponding author.

## Ethics statement

The studies involving humans were approved by Comité Científico de la Universidad Católica de la Santísima Concepción. The studies were conducted in accordance with the local legislation and institutional requirements. The participants provided their written informed consent to participate in this study.

## Author contributions

CC-D: Conceptualization, Data curation, Funding acquisition, Investigation, Methodology, Project administration, Supervision, Visualization, Writing – original draft, Writing – review & editing. AV-C: Conceptualization, Data curation, Formal analysis, Investigation, Writing – review & editing. SU-L: Data curation, Formal analysis, Software, Writing – review & editing. JO: Conceptualization, Supervision, Writing – review & editing. VD-G: Conceptualization, Methodology, Writing – review & editing.
